# Construction and Expression Analysis of the *bin ^3xOllas^* Tool Line

**DOI:** 10.17912/micropub.biology.001958

**Published:** 2026-01-09

**Authors:** Mengyuan Yi, Hannah Sonnenberg, Vimala Anthonydhason, Ruth H. Palmer

**Affiliations:** 1 Department of Medical Biochemistry and Cell Biology, Institute of Biomedicine at the Sahlgrenska Academy, University of Gothenburg, Gothenburg, Västra Götaland, Sweden; 2 Centre for Tumour Microenvironment, Barts Cancer Institute, Queen Mary University of London, London, England, United Kingdom

## Abstract

Precise detection of endogenous protein expression is essential for understanding gene function *in vivo* . We generated a *Drosophila* knock-in line inserting a 3×Ollas tag at the C-terminus of the endogenous *biniou* ( *bin* ) locus, enabling specific visualization of the Bin transcription factor. The *bin ^3xOllas^* allele faithfully recapitulates native expression in the visceral mesoderm and does not disrupt Bin function. Integrating single-cell RNA-sequencing data, we further identified Bin expression in adult reproductive tissues, including ovarian escort cells and testis hub and cyst cells. This allele provides a robust tool for studying Bin function and dynamics.

**Figure 1. Generation and expression patterns of the endogenous Bin 3xOllas Drosophila line:**
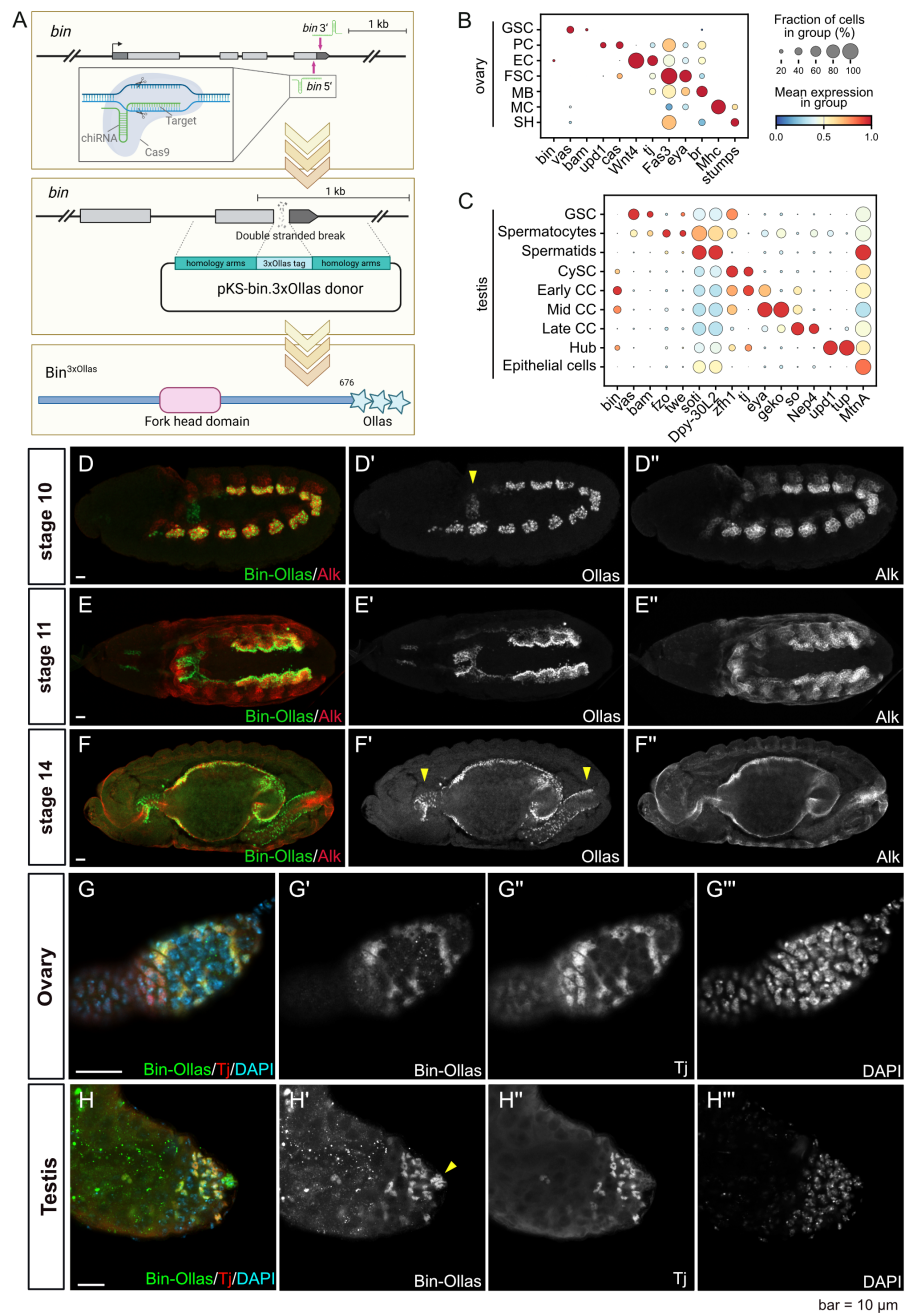
**(A)** Schematic outlining the CRISPR/Cas9-mediated knock-in strategy employed to generate the *bin ^3×Ollas^* endogenous tagged allele. **(B–C)** scRNA-seq–based *bin* expression profiles. &nbsp;(B) In the ovary, *bin* mRNA is enriched in escort cells (EC). &nbsp;(C) In the testis, *bin* mRNA is predominantly expressed in cyst cells (CC) and hub cells (Hub). Major cell populations are annotated based on selected marker genes. Color gradient indicates normalized expression, dot size indicates the percentage of expressing cells. GSC: Germline Stem Cells, PC: Polar Cells, EC: Escort Cells, FSC: Follicle Stem Cells, MB: Main Body Follicle Cells, MC: Muscle Cells, SH: Sheath cells, CySC: Cyst Stem Cells, CC: Cyst cells, Hub: Hub Cells. Single cell data analysis was performed on publicly available ovary (Slaidina et al., 2020) and testis (Witt et al., 2019) datasets. **(D–F)** Embryonic expression of Bin ^3×Ollas^ . Scale bar corresponds to 10 μm. (D) At stage 10, Bin ^3×Ollas^ protein is detected in visceral mesoderm (VM) clusters and co-localizes with Alk. Arrowhead marks caudal VM. (E) At stage 11, Bin ^3×Ollas^ protein expression is observed throughout the VM and appears stronger in founder cells (FC). (F) At stage 14, Bin ^3×Ollas^ protein is present throughout the gut primordia, including foregut, midgut, and hindgut. Arrowheads mark foregut and hindgut. **(G–H)** Adult gonad Bin ^3×Ollas^ expression. Scale bar corresponds to 10 μm. (G) In the ovary, Bin ^3×Ollas^ marks escort cells and co-localizes with the escort cell marker Tj. (H) In the testis, Bin ^3×Ollas^ is detected in hub cells and cyst cells, also co-localizing with Tj. Arrowhead marks hub cells.

## Description

The FoxF family transcription factor *biniou (bin)* is essential for visceral mesoderm (VM) specification and midgut morphogenesis during *Drosophila* embryogenesis. Together with Bagpipe (Bap) (Azpiazu and Frasch 1993) and Alk (Lorén et al., 2001), Bin expression is an early marker of the embryonic VM and is employed as a VM marker (Zaffran et al., 2001; Jakobsen et al., 2007). Downstream of Bin lies a broad transcriptional program required for VM development and myogenesis, including *bap* (which participates in a positive feedback loop), *dpp* , *βTub60D* , *hand* , and many other VM- and muscle-related genes (Lorén et al., 2001; Zaffran et al., 2001; Zaffran and Frasch 2002; Jakobsen et al., 2007; Popichenko et al., 2007; Anllo and DiNardo 2022).

To enable direct detection of endogenous Bin protein, we developed a CRISPR/Cas9-mediated *bin ^3xOllas^* knock-in allele, which introduces a C-terminal 3xOllas epitope tag ( Figure 1A ). The Ollas tag (14 amino acids) is a synthetic, highly specific epitope with minimal background in *Drosophila* , thus providing a clean and reliable tool for *in vivo* immune detection (Park et al., 2008). Importantly, while *bin* mutants are embryonic lethal (Zaffran et al., 2001), homozygous *bin ^3xOllas^* flies were viable and fertile, and no developmental abnormalities were observed, indicating that the tag does not disrupt Bin function. Using this line, we first confirmed that Bin ^3×Ollas^ faithfully reflects endogenous Bin distribution in the embryonic VM. At stage 10, discrete clusters of Bin *^3×Ollas^* –positive cells corresponding to early VM precursors were observed ( Figure 1D ). By stage 11, these clusters extended both anteriorly and posteriorly to form a continuous Bin-positive VM domain, Bin ^3xOllas^ expression appears enriched in founder cells ( Figure 1E ). These founder cells subsequently fuse with neighboring fusion-competent myoblasts to form multinucleate visceral muscle. By stage 14, Bin ^3×Ollas^ -positive cells were observed in the entire muscle layer surrounding the midgut. A strong Bin ^3xOllas^ signal was also consistently observed in the foregut and hindgut mesoderm ( Figure 1F ). These results demonstrate that the *bin ^3×Ollas^* line faithfully reports endogenous spatial and temporal expression dynamics of *bin* during embryonic VM development without impacting its transcription factor function, providing a reliable tool for assessing potential expression in additional tissues.

The availability of this line, combined with publicly available single-cell RNA-seq datasets (Li et al., 2022), prompted a re-examination of bin expression beyond embryonic tissues. scRNA-seq profiles revealed unexpected *bin* expression in adult gonads. In the ovary, *bin* transcripts were specifically enriched in escort cells (EC), a somatic population surrounding early germline cells ( Figure 1B ). In the testis, bin expression was detected in hub cells and cyst cell lineages, with the strongest signal in early cyst cells ( Figure 1C ). These transcriptomic data suggested previously unrecognized roles for Bin in somatic support cell populations.

Guided by this transcriptomic analysis, we examined Bin ^3xOllas^ expression in adult gonads. In the ovary, Bin ^3xOllas^ localized specifically to EC within the germarium ( Figure 1G ), consistent with both the scRNA-seq results and a recent study showing that Bin maintains EC identity and shapes the GSC-supportive niche by modulating BMP signaling (Tu et al., 2021). Similarly, in the testis, Bin ^3xOllas^ was detected in hub cells and in cyst stem cell (CySC) and early cyst cell populations, with weaker expression in late cyst cells ( Figure 1H ), closely matching the transcriptomic anaylses. Although little is known about Bin function in cyst cells, a recent embryonic study has shown that Bin activity in the VM generates signals such as Slit and FGF to maintain testis precursor niche polarization (Anllo and DiNardo 2022). The presence of Bin protein in adult somatic gonadal cells therefore raises the possibility of direct roles for Bin within the testis itself, a question that remains open for future investigation.

Together, these findings establish the *bin ^3×Ollas^* line as a reliable and versatile tool for investigating Bin function in diverse developmental contexts and uncovers a previously unappreciated adult gonadal expression of Bin with potential implications for somatic control of germ cell development.

## Methods

**Single-cell RNA-seq analysis of *Drosophila* gonads**

Single-cell RNA-seq data from *Drosophila melanogaster* testis and ovary were obtained from the Fly Cell Atlas (https://cloud.flycellatlas.org) and analyzed using Scanpy in Python. Cell annotations were harmonized to broader biological categories using published references (Slaidina et al., 2020 for ovary; Witt et al., 2019 for testis). In ovary, cells were grouped into: Germline Stem Cells (GSC), Polar Cells (PC), Escort Cells (EC), Follicle Stem Cells (FSC), Main Body Follicle cells (MB), Muscle Cells (MC), and Sheath Cells (SH). In testis, cells were grouped into: Germline Stem Cells (GSC), spermatocytes, spermatids, Cyst Stem Cells (CySC), Early Cyst Cells (Early CC), Mid Cyst Cells (Mid CC), Late Cyst Cells (Late CC), Hub Cells (Hub), and Epithelial Cells. Marker genes for each population were curated from the literature, and dot-plot visualizations were generated to show scaled expression of lineage-specific markers across gonadal cell types.

**Generation of *bin ^3xOllas^* Knock-in Fly Allele**

The *bin ^3×Ollas^* allele was generated following the CRISPR/Cas9 protocol previously used to generate *bap ^HA^* (Wolfstetter et al., 2025). Briefly, a *bin.3×Ollas* *pBluescript* *II KS (-)* (GenScript) donor was constructed containing 782 bp upstream and 776 bp downstream homology arms flanking a sequence encoding three tandem 14 amino acid Ollas tags (SGFANELGPRLMGK–SGFANELGPRLMGK–SGFANELGPRLMGK). The *pU6-BbsI-chiRNA* gRNA expression vector (Addgene) carrying two CRISPR target sites (5′-TAGGCCGGCTTGCGATCAATGGG-3′ and 5′-ATGCACGCCATCCCAAGTTGAGG-3′) was injected together with the *bin.3xOllas* donor into *y ^1^ M{vas-Cas9}ZH-2A* embryos (Bloomington 51323) by BestGene Inc.. The *bin ^3×Ollas^* allele was confirmed by Sanger sequencing (GATC services, Eurofins).

**Sample Preparation, Immunofluorescence, and Imaging**

All washes were performed with PBS containing 0.3% NP-40 (wash solution). Embryos were collected over a 6–12 h period and dechorionated in 50% bleach (2.5% NaClO) for 3 minutes, then rinsed thoroughly with water. Fixation was carried out in glass vials containing 2 mL of heptane and 2 mL of 4% formaldehyde in PBS, shaken at room temperature (200 rpm) for 40 minutes. After removing the lower formaldehyde phase, 2 mL of methanol was added, and the vial was vigorously shaken for 1 minute to remove the vitelline membrane. Embryos were washed in 100% methanol and stored at –20 °C. Before staining, embryos were rehydrated through 50% methanol/50% wash solution followed by 100% wash solution.

Testes and ovaries were dissected from adult flies anesthetized with CO _2_ in cold PBS. Ovarioles were carefully separated using fine needles. Tissues were fixed in 4% formaldehyde for 15 minutes, followed by three washes (3 × 15 minutes) in wash solution.

For immune staining, samples were blocked in 5% normal goat serum (NGS) in wash solution for 30 minutes at room temperature. Primary antibodies were applied overnight at 4 °C. After three washes, samples were incubated with Alexa Fluor-conjugated secondary antibodies (Thermo Fisher) for 2 hours at room temperature in the dark. DAPI was used to label nuclei. After three washes, all samples were mounted in Fluoromount-G (SouthernBiotech). Primary antibodies employed were rat anti-Ollas (MAb L2, Novus) at 1:20, rabbit anti-Alk (Boy) at 1:750 (Lorén et al., 2003), and guinea pig anti-Traffic jam (Tj; gift from D. Godt) at 1:5000 (Li et al., 2003; Gunawan et al., 2013). Secondary antibodies employed were from Jackson Immunoresearch and were employed at 1:500. All samples were imaged using a Zeiss Axio Imager Z2 with LSM 800.

## Reagents

**Table:** 

**Strain genotype**	**Source**	**Identifier**
* y ^1^ M{vas-Cas9}ZH-2A *	Bloomington	BDSC: 51323
* bin ^3xOllas^*	This study	&nbsp;
**Plasmid genotype**	**Source**	**Identifier**
pU6-BbsI-chiRNA	Addgene	45946
pBlueScript-II-KS(-)-bin.3xOllas donor	This study	&nbsp;
**Antibody**	**Source**	**Identifier**
rat anti-Ollas&nbsp;&nbsp;&nbsp;	Novus	MAb L2
rabbit anti-Alk	Palmer lab (Lorén et al., 2003)	Boy
guinea pig anti-Tj	Gift from D. Godt (Li et al., 2003; Gunawan et al., 2013)	&nbsp;
Fluorophore coupled secondary antibodies	Jackson Immunoresearch	706-606-148 &nbsp; &nbsp;&nbsp;Gp647 111-546-144&nbsp;&nbsp;&nbsp;&nbsp; Rb488 715-166-151&nbsp;&nbsp;&nbsp;&nbsp; MCy3
